# Comparative safety of imeglimin versus sitagliptin in Japanese patients with type 2 diabetes: A pharmacy-based retrospective cohort study

**DOI:** 10.1371/journal.pone.0359960

**Published:** 2026-10-07

**Authors:** Nao Suzuki, Nobuhiro Ooba, Rie Nakajima, Kasumi Takabatake, Masahiro Saita, Eiji Kawakami, Michiyo Kawana, Daiki Watanabe, Hajime Hashiba

**Affiliations:** 1 Department of Pharmacoepidemiology, Nihon University School of Pharmacy, Funabashi, Chiba, Japan; 2 Department of Pharmacy Practice in Primary Care, Nihon University School of Pharmacy, Funabashi, Chiba, Japan; 3 Japan Pharmaceutical Association, Shinjuku-ku, Tokyo, Japan; 4 Department of Pharmaceutical Sciences, Teikyo University School of Pharmacy, Itabashi-ku, Tokyo, Japan; Al-Azhar University / King Khalid University, EGYPT

## Abstract

Clinical evidence supports the safety and efficacy of imeglimin, a new oral antidiabetic medication that increases insulin secretion and reduces insulin resistance. However, comparative real-world safety data with dipeptidyl peptidase-4 inhibitors, widely used in Japan, remain limited. This retrospective, pharmacy-based cohort study compared the incidence of adverse events (gastrointestinal events and hypoglycemia) among patients newly prescribed imeglimin or sitagliptin in real-world settings. Using a new-user design, we analyzed the 2022–2024 community pharmacy data across Japan, including 1,140 and 990 new imeglimin and sitagliptin users, respectively. Primary outcome (incidence of treatment-related events classified using Medical Dictionary for Regulatory Activities terminology) was evaluated using Cox proportional hazards models. Unadjusted and multivariable-adjusted hazard ratios (aHRs) for events during a 4-month follow-up were estimated. At least one adverse event was reported among 31% of imeglimin users versus 22% of sitagliptin users. Hypoglycemia risk differed non-significantly between groups (aHR: 0.66; 95% confidence interval [CI]: 0.20–2.22). However, imeglimin was associated with substantially higher risks of gastrointestinal events than sitagliptin (aHR: 5.44; 95% CI: 3.44–8.61), particularly nausea (aHR: 15.34; 95% CI: 3.50–67.23), diarrhea (aHR: 7.90; 95% CI: 2.54–24.62), and abdominal discomfort (aHR: 6.02; 95% CI: 1.47–24.58). Sensitivity analyses in patients receiving monotherapy yielded increased risk (hazard ratio (HR): 5.36; 95% CI: 2.63–10.92). Imeglimin use was associated with a higher incidence of gastrointestinal events than sitagliptin in Japanese clinical practice, closely approaching our prespecified threshold for clinical significance. Conversely, no significant difference was observed in hypoglycemia risk, although this finding should be interpreted with caution owing to the limited number of events. Involving community pharmacists allowed for active monitoring and identification of events that are often underreported in large-scale claims databases. Clinicians should prioritize gastrointestinal monitoring during initial stages of imeglimin therapy, particularly in older patients with complex treatment regimens.

## Introduction

Diabetes is characterized by hyperglycemia and is diagnosed using HbA1c and plasma glucose levels [1,2]. Diabetes represents a major risk factor for microvascular and macrovascular complications [3], making effective glycemic management essential to reduce these risks [4,5]. In 2024, the global prevalence of diabetes was estimated at 589 million individuals, accounting for 11.1% of adults aged 20–79 years [6]. In Japan, approximately 10.8 million people are affected [6]. With 29.3% of the Japanese population aged 65 years and older [7] and diabetes incidence increasing with age, the number of affected individuals is expected to rise further [8]. Older patients often present with multiple comorbidities, including hypertension and dyslipidemia, which increase the risk of renal failure and cardiovascular disease [9].

International guidelines, such as the American Diabetes Association Standards of Care, recommend therapies, including metformin, sodium–glucose cotransporter-2 (SGLT2) inhibitors, and glucagon-like peptide-1 (GLP-1) receptor agonists, tailored to a patient’s pathophysiology [10]. In the United Kingdom, treatment guidelines advocate biguanides, particularly metformin, as first-line therapy for type 2 diabetes [11]. In contrast, dipeptidyl peptidase-4 (DPP-4) inhibitors remain the most frequently prescribed glucose-lowering agents in Japan, followed by biguanides and SGLT2 inhibitors [12]. This pattern likely reflects their efficacy in lowering HbA1c, low hypoglycemia risk, and high treatment persistence among Japan’s predominantly older population with diabetes [12–14]. DPP-4 inhibitors are generally not associated with increased gastrointestinal symptoms [15,16] or digestive system infections [17]. Previous trials, including meta-analyses, have reported a low risk of hypoglycemia with sitagliptin, a widely used DPP-4 inhibitor [14,16].

Imeglimin is a novel therapeutic agent that lowers blood glucose by enhancing insulin secretion and improving insulin sensitivity [18]. It was first approved for clinical use in Japan, with further development and regulatory approvals ongoing in other Asian countries. According to the Japanese Risk Management Plan, hypoglycemia and gastrointestinal symptoms are identified as notable potential risks [19]. In line with this, randomized controlled trials (RCTs) have demonstrated that while imeglimin effectively reduces HbA1c by approximately 0.46% [20], it is also associated with adverse effects such as diarrhea (5.4%), gastritis (2.7%), abdominal discomfort (2.7%), and hypoglycemia (2.7%) [21].

Despite its clinical approval, comparative real-world data on hypoglycemia and gastrointestinal events between imeglimin and widely used DPP-4 inhibitors, especially in older populations, remain limited. Therefore, in this study, we aimed to evaluate these outcomes among new users of imeglimin and sitagliptin using a retrospective cohort design based on primary community pharmacy data. To distinguish clinical from statistical significance, a 10% absolute difference in the incidence of gastrointestinal symptoms was prespecified as the minimum clinically relevant threshold. In the absence of a universally established standard for this outcome [22], this threshold was chosen as a pragmatic benchmark to guide potential changes in prescribing practices, informed by the known safety profiles of oral hypoglycemic agents. By conducting this analysis, we sought to characterize the comparative safety of these therapies in a real-world setting and provide clinically relevant evidence to support treatment selection.

## Materials and methods

### Study design and participants

This retrospective cohort study employed a new-user design [23] to compare the incidence of events between users of imeglimin and sitagliptin. Sitagliptin was selected as the sole active comparator because DPP-4 inhibitors are the most frequently prescribed first-line oral antidiabetic agents in Japan [12]. Furthermore, sitagliptin has the highest prescription rate among the DPP-4 inhibitors in Japanese real-world clinical practice [24]. From February 2023 through February 2025, questionnaires were distributed by the Japan Pharmaceutical Association (JPA) to 50,710–53,350 affiliated pharmacies. In accordance with methodologies of previous studies [25–27], these questionnaires were used to identify new users of imeglimin and sitagliptin and record baseline characteristics and event data. Survey participation included 4,795 pharmacies in 2022, 4,545 in 2023, and 4,517 in 2024.

Consistent with established protocols [25–27], community pharmacists identified new users of the study drugs. Patients prescribed either drug in September of each study year (2022–2024) were initially selected. “New users” were defined as individuals who had visited the pharmacy at least once prior to March of the same year but had no recorded use of the target drug during the 6-month washout period from March to August. Patients who were prescribed the drug in September but had no subsequent pharmacy visits were excluded. In addition, patients who switched from imeglimin to sitagliptin, or vice versa, during the observation period were excluded to ensure that any adverse events could be appropriately attributed to the specific medication. The final study population comprised pooled data from newly identified users across the 3-year study period.

### Data collection and follow-up

The observation period for each year spanned 4 months, from September 1 to December 31. Community pharmacists played a critical role in clinical practice by explaining prescribed medications to patients and actively monitoring treatment adherence and the incidence of adverse drug reactions. The time scale for the survival analysis was days. The event dates were determined from pharmacy records. Pharmacists monitored and recorded the occurrence of adverse events through direct patient interviews conducted at each pharmacy visit upon presentation of a prescription, rather than at fixed monthly intervals.

Pharmacists reviewed pharmacy records to identify relevant events within this timeframe. Baseline data (collected during the 6 months preceding therapy initiation) included the drug class, start date, daily dosage, smoking and alcohol status, use of over-the-counter (OTC) medications, comorbidities, concomitant therapies, and history of hospitalization. Information regarding hospitalization history was obtained from pharmacy records, including information collected through patient interviews and medication notebooks. All data were entered into a predesigned web-based reporting form. Specifically, the survey was conducted at each pharmacy over a one-month period in February of 2023, 2024, and 2025. Subsequently, the authors had access to the compiled, anonymized data for this research from March 1, 2023 to March 5, 2025.

### Definition of events

Pharmacists identified events, including adverse drug reactions, from pharmacy records that were evaluated for a potential causal relationship with the medication [28]. Data regarding the discontinuation of the target drug or initiation of additional medications were collected to support event assessment. All reported events were coded using the Medical Dictionary for Regulatory Activities, version 27.0. First, low-level terms were assigned to each event and subsequently converted to preferred terms for analysis [29].

### Statistical analysis

Summary statistics were calculated for sex, age, comorbidities, concomitant medications, and hospitalization history. There were no missing data for the baseline characteristics, including smoking status, as these variables were routinely collected and verified by pharmacists through direct patient interviews during medication counseling. Baseline characteristics between the two groups were assessed using standardized mean differences (SMDs), with an SMD > 0.10 indicating a clinically meaningful imbalance. Variables showing notable differences were included as covariates in the multivariable analysis. Given the limited number of outcome events, covariates for the multivariable models were selected using a stepwise approach to reduce the risk of model overfitting while accounting for confounding. Established risk factors, including use of antidiabetic agents other than insulin and insulin use as indicators of diabetes severity, were retained in the final multivariable model regardless of significance.

The incidence of events, including treatment discontinuation, was calculated for each group. Events reported in ≥5 cases, as well as all serious events, were analyzed in detail. As presented in the tables, incidence proportions, absolute risk differences, and corresponding 95% confidence intervals (CIs) were calculated for adverse events and add-on medications. Additionally, Kaplan-Meier curves were generated to estimate the cumulative incidence of the primary outcome over the follow-up period. HRs with 95% CIs were estimated using Cox proportional hazards models and presented as unadjusted, age- and sex-adjusted, and multivariable-adjusted. The observation period was defined from drug initiation to the earliest of the following: event occurrence, drug switch or discontinuation, addition of an alternative glucose-lowering agent, last patient visit, or December 31 of the respective year. Adjustment factors included age, sex, smoking status, alcohol consumption, hypertension, dyslipidemia, hyperuricemia, myocardial infarction, heart failure, cancer, hospitalization history, and concomitant medications (antihypertensives; glucose-lowering agents excluding insulin; insulin; lipid-lowering drugs; and hypouricemic agents).

As part of sensitivity analyses, the risk of gastrointestinal symptoms was evaluated in a subpopulation of monotherapy users who were not receiving other glucose-lowering agents at baseline, aside from the study drugs. Furthermore, because several outcomes had a very low number of events per variable, sensitivity analyses were performed using Firth’s penalized likelihood Cox regression for both adverse events and add-on medications. This approach was employed to address potential small-sample bias and improve the stability of the HR estimates. The results of these sensitivity analyses are presented in S1 and S2 Tables.

Formal sample size calculations were not performed because all new users of imeglimin or sitagliptin during the observation period were included, minimizing selection bias. Nevertheless, the estimated sample size was 1,811, assuming a 2.5% event incidence in the control group, an α = 0.05, power = 0.8, and HR = 2.0. Statistical significance was defined as p < 0.05. All analyses were conducted using SAS version 9.4 (SAS Institute Inc., Cary, NC, USA).

### Ethical considerations

This study adhered to the tenets of the Declaration of Helsinki and was approved by the Ethics Review Committee of the JPA (approval numbers: 2022-001-01, 2023-003-01, and 2024-001-01). The need for individual informed consent was waived because all study data were fully anonymized.

## Results

### Patients’ characteristics

In September 2022, 2023, and 2024, a total of 3,621 patients were prescribed imeglimin and 24,046 patients were prescribed sitagliptin. During patient selection, 437 patients in the imeglimin group and 224 patients in the sitagliptin group who switched between imeglimin and sitagliptin during the observation period were excluded. Finally, 1,140 (31%) imeglimin users and 990 (4%) sitagliptin users met the criteria for “new users” and were included in the study. The mean age was 66.9 years in the imeglimin group and 69.9 years in the sitagliptin group (Table 1). Average daily doses were 1,619.4 mg for imeglimin and 46.2 mg for sitagliptin. Mean observation periods were similar, at 99 days for imeglimin and 101 days for sitagliptin. Notable baseline differences, reflected by absolute SMDs > 0.1, were observed for age, dyslipidemia, concomitant use of other antidiabetics or insulin, lipid-lowering drugs, and hospitalization history (Table 1).

**Table 1:** Baseline characteristics.

Characteristic	Imeglimin (n = 1,140)	Sitagliptin (n = 990)	Standardized mean difference
**Age (years), mean (SD)**	66.9 (13.8)	69.9 (13.5)	−0.2182
**Male sex, n (%)**	697 (61.1)	574 (58.0)	0.0644
**Lifestyle factor, n (%)**			
**Smoking**	170 (14.9)	148 (14.9)	−0.0010
**Alcohol consumption**	284 (24.9)	258 (26.0)	−0.0264
**OTC drug use**	26 (2.3)	23 (2.3)	−0.0028
**Comorbidity, n (%)**			
**Hypertension**	662 (58.1)	594 (60.0)	−0.0393
**Dyslipidemia**	670 (58.8)	416 (42.0)	0.3398
**Myocardial infarction**	29 (2.5)	24 (2.4)	0.0077
**Heart failure**	45 (3.9)	30 (3.0)	0.0500
**Peptic ulcer**	109 (9.6)	99 (10.0)	−0.0148
**Asthma**	37 (3.2)	50 (5.1)	−0.0906
**Cancer**	47 (4.1)	54 (5.5)	−0.0624
**Hyperuricemia**	94 (8.2)	81 (8.2)	0.0023
**History of hypoglycemia**	8 (0.7)	1 (0.1)	0.0951
**Concomitant medications, n (%)**			
**Antihypertensives**	678 (59.5)	613 (61.9)	−0.0501
**Antidiabetics, except insulin**	976 (85.6)	399 (40.3)	1.0625
**Insulin**	222 (19.5)	58 (5.9)	0.4182
**Lipid-lowering drugs**	681 (59.7)	447 (45.1)	0.2952
**Hypouricemic agents**	109 (9.6)	93 (9.4)	0.0057
**Hypnotics**	126 (11.1)	116 (11.7)	−0.0209
**Antiulceratives**	232 (20.4)	234 (23.6)	−0.0794
**History of hospital admission**	14 (1.2)	48 (4.8)	−0.2121

SD, standard deviation; OTC, over-the-counter

Treatment was discontinued in 9.8% (112/1,140) of imeglimin users and 6.7% (66/990) of sitagliptin users, yielding a crude risk ratio (RR) of 1.47 (95% CI: 1.09–2.00). Drug switching occurred in 4.8% (55 patients) of the imeglimin group and 4.0% (40 patients) of the sitagliptin group, with a RR of 1.19 (95% CI: 0.79–1.79).

### Events

The incidence of at least one reported event was 31% (351 cases) for imeglimin and 22% (220 cases) for sitagliptin (crude RR: 1.39; 95% CI: 1.20–1.60). Among imeglimin users, the most frequently reported adverse events were gastrointestinal symptoms (12.8%), decreased therapeutic response (6.1%), and nausea (3.6%; Table 2). Notably, imeglimin use was associated with a significantly higher risk of nausea (adjusted HR [aHR]: 15.34; 95% CI: 3.50–67.23), diarrhea (aHR: 7.90; 95% CI: 2.54–24.62), elevated blood glucose (aHR: 4.57; 95% CI: 1.20–17.34), and abdominal discomfort (aHR: 6.02; 95% CI: 1.47–24.58). This association may reflect confounding by indication or temporary worsening of glycemic control during treatment discontinuation or medication switching rather than a direct pharmacological effect of imeglimin. Additionally, events such as vomiting (1.4%), soft stools (1.1%), and dyspepsia (0.4%) were reported exclusively in the imeglimin group. The Kaplan-Meier curves illustrating the cumulative incidence of gastrointestinal events during follow-up are presented in Fig 1.

**Table 2:** Incidence of common events among imeglimin and sitagliptin users.

Common event	Imeglimin (n = 1,140)	Sitagliptin (n = 990)	Absolute risk difference, % (95% confidence interval)	Hazard ratio (95% confidence interval)		
				Unadjusted	Age–sex-adjusted	Multivariable-adjusted^a^
**Gastrointestinal symptoms**	146 (12.8%)	27 (2.7%)	9.8 (7.7–12.0)	4.84 (3.21–7.30)	4.92 (3.26–7.43)	5.44 (3.44–8.61)
**Nausea**	41 (3.6%)	2 (0.2%)	3.4 (2.3–4.7)	18.14 (4.39–74.99)	17.81 (4.30–73.74)	15.34 (3.50–67.23)
**Diarrhea**	34 (3.0%)	4 (0.4%)	2.6 (1.5–3.8)	7.54 (2.68–21.24)	7.26 (2.57–20.51)	7.90 (2.54–24.62)
**Constipation**	14 (1.2%)	10 (1.0%)	0.2 (−0.8–1.2)	1.24 (0.55–2.78)	1.34 (0.59–3.01)	1.35 (0.52–3.53)
**Abdominal discomfort**	12 (1.1%)	3 (0.3%)	0.8 (0.01–1.6)	3.56 (1.00–12.61)	3.80 (1.07–13.57)	6.02 (1.47–24.58)
**Gastrointestinal disorder**	8 (0.7%)	3 (0.3%)	0.4 (−2.8–1.1)	2.35 (0.62–8.87)	2.27 (0.60–8.65)	2.27 (0.49–10.47)
**Abdominal distension**	7 (0.6%)	2 (0.2%)	0.4 (−2.1–1.1)	3.09 (0.64–14.85)	3.25 (0.67–15.75)	4.87 (0.85–27.86)
**Upper abdominal pain**	2 (0.2%)	3 (0.3%)	−0.1 (−0.7–0.4)	0.58 (0.10–3.49)	0.57 (0.09–3.47)	0.82 (0.09–7.50)
**Decreased therapeutic response**	70 (6.1%)	43 (4.3%)	1.8 (−0.1–3.7)	1.44 (0.99–2.11)	1.32 (0.90–1.93)	1.15 (0.74–1.78)
**Inadequate control of diabetes mellitus**	23 (2.0%)	14 (1.4%)	0.6 (−0.1–1.8)	1.45 (0.75–2.82)	1.43 (0.73–2.80)	1.16 (0.534–2.49)
**Increased blood glucose**	19 (1.7%)	3 (0.3%)	1.4 (0.5–2.3)	5.59 (1.65–18.87)	5.40 (1.59–18.33)	4.57 (1.20–17.34)
**Increased glycosylated hemoglobin**	14 (1.2%)	9 (0.9%)	0.3 (−0.6–1.3)	1.38 (0.60–3.19)	1.28 (0.55–2.99)	1.34 (0.50–3.59)
**Therapy non-responder**	14 (1.2%)	3 (0.3%)	0.09 (0.2–1.8)	4.11 (1.18–14.30)	3.72 (1.06–13.03)	3.90 (0.96–15.78)
**Pre-existing condition improved**	6 (0.5%)	18 (1.8%)	−1.3 (−2.4–-0.4)	0.29 (0.12–0.74)	0.29 (0.11–0.74)	0.25 (0.09–0.70)
**Decreased glycosylated hemoglobin**	6 (0.5%)	5 (0.5%)	0.02 (−0.71–0.70)	1.07 (0.33–3.50)	1.13 (0.34–3.71)	1.05 (0.25–4.47)
**Decreased appetite**	16 (1.4%)	3 (0.3%)	1.1 (0.3–2.0)	4.67 (1.36–16.02)	5.33 (1.55–18.33)	2.89 (0.74–11.35)
**Increased blood pressure**	14 (1.2%)	11 (1.1%)	0.1 (−0.9–1.1)	1.12 (0.51–2.46)	1.17 (0.53–2.60)	1.31 (0.52–3.32)
**Pruritus**	5 (0.4%)	10 (1.0%)	−0.6 (−1.5–0.2)	0.44 (0.15–1.28)	0.47 (0.16–1.39)	0.60 (0.16–2.23)
**Hypertension**	4 (0.4%)	6 (0.6%)	−0.3 (−1.0–0.4)	0.59 (0.17–2.09)	0.56 (0.16–2.00)	0.57 (0.12–2.63)
**Dizziness**	8 (0.7%)	5 (0.5%)	0.2 (−0.6–0.9)	1.40 (0.46–4.29)	1.58 (0.52–4.85)	1.55 (0.41–5.89)
**Hypoglycemia**	7 (0.6%)	7 (0.7%)	−0.1 (−0.9–0.7)	0.87 (0.31–2.49)	0.89 (0.31–2.56)	0.66 (0.20–2.22)
**Insomnia**	5 (0.4%)	5 (0.5%)	−0.1 (−0.8–0.6)	0.87 (0.25–3.01)	0.86 (0.25–3.01)	0.68 (0.16–2.22)
**Increased blood cholesterol**	4 (0.4%)	6 (0.6%)	−0.3 (−1.0–0.4)	0.59 (0.17–2.08)	0.60 (0.17–2.13)	0.60 (0.13–2.66)

^a^Adjusted for age; male sex; smoking status; alcohol; use of antihypertensives, antidiabetic drugs (except insulin), insulin, lipid-lowering drugs, hypouricemic agents, and steroids (except topical steroids); hypertension; dyslipidemia; myocardial infarction; chronic heart failure; cancer; hyperuricemia; and hospitalization.

**Fig 1:**
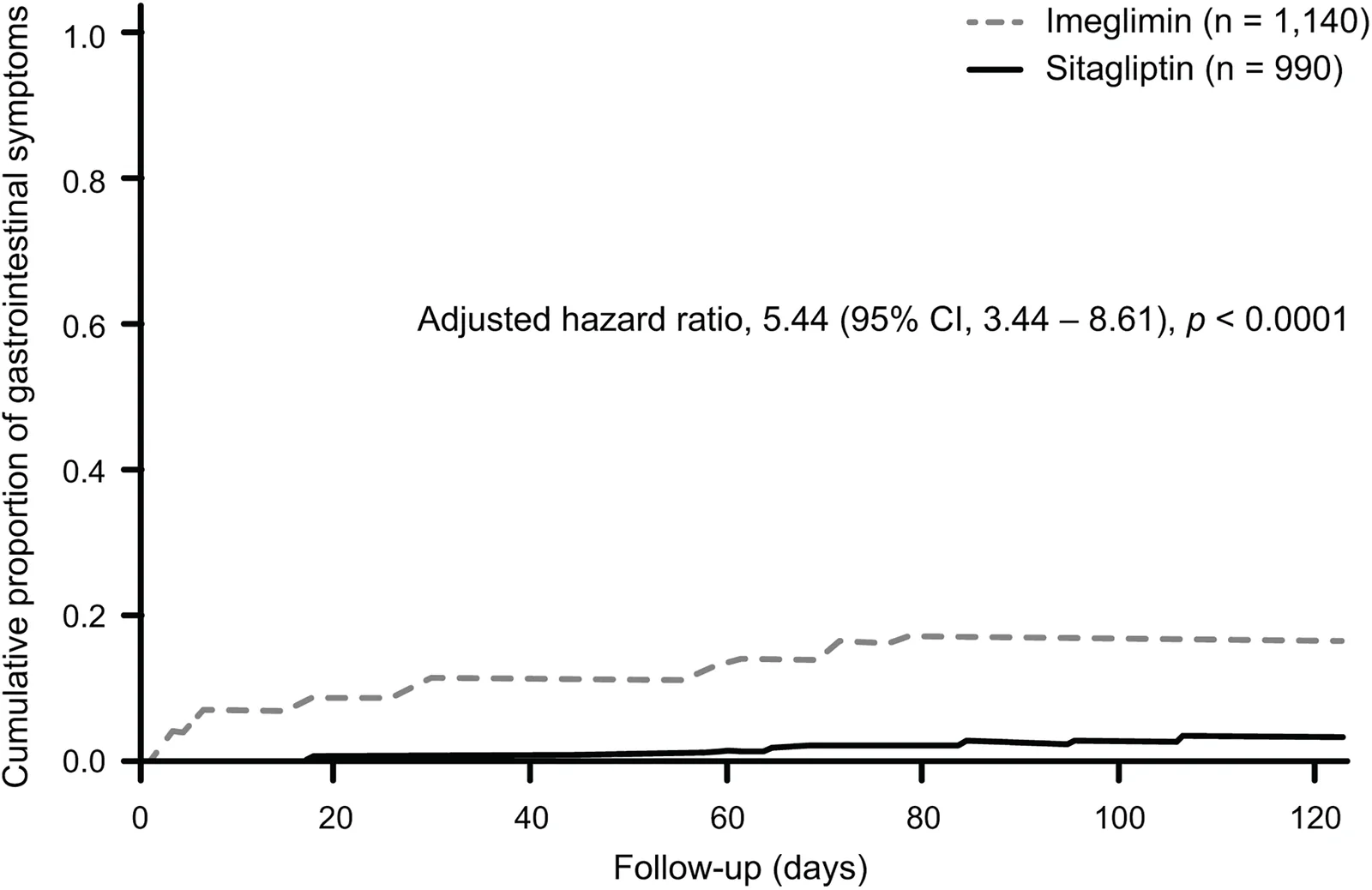
Kaplan-Meier curves for the cumulative incidence of gastrointestinal events. The curves show the cumulative incidence of gastrointestinal symptoms in patients newly prescribed imeglimin (dashed gray line) compared with that in patients prescribed sitagliptin (solid black line) during the follow-up period. The hazard ratio, 95% confidence interval, and p-value displayed in the figure were derived from the multivariable Cox proportional hazards model.

Among sitagliptin users, the most common events were decreased therapeutic response (4.3%), improvement in pre-existing conditions (1.8%), and inadequate control of diabetes mellitus (1.4%). The rate of improvement of pre-existing conditions was higher in the sitagliptin group than in the imeglimin group (aHR: 0.25; 95% CI: 0.09–0.70; Table 2). To minimize potential confounding effects of concomitant antidiabetic medications, we performed sensitivity analyses restricted to patients receiving monotherapy (imeglimin, n = 114; sitagliptin, n = 561). In this monotherapy subpopulation, 19 gastrointestinal events occurred in the imeglimin group and 15 among sitagliptin users. The sensitivity analyses showed an elevated risk of gastrointestinal events among imeglimin users (aHR: 5.36; 95% CI: 2.63–10.91), with an absolute risk difference of 13.4% (95% CI: 7.6%–21.2%). Similarly, the aHR (5.20; 95% CI: 2.55–10.61) estimated using Firth’s penalized likelihood approach for Cox regression was comparable to the primary analysis. The results of the sensitivity analyses using Firth’s penalized likelihood approach for all events were also consistent with the primary analysis (S1 Table).

### Add-on medications

Among imeglimin users, 25.3% (288 patients) had new medications added, compared with 31.8% (315 patients) of sitagliptin users (crude RR: 0.79; 95% CI: 0.69–0.91). Table 3 shows that the most common add-on medications among imeglimin users were non-insulin antidiabetic drugs (9.5%) and insulin or GLP-1 receptor agonists (2.6%). Imeglimin users showed a tendency toward higher use of antinauseants and non-insulin glucose-lowering agents, although this difference was not significant (Table 3). The results of sensitivity analyses using Firth’s penalized likelihood approach for all events were consistent with those of the primary analysis (S2 Table).

**Table 3:** Incidence of any add-on medication use among imeglimin and sitagliptin users.

Drug	Imeglimin (n = 1,140)	Sitagliptin (n = 990)	Absolute risk difference, % (95% confidence interval)	Hazard ratio (95% confidence interval)		
				Crude	Age–sex-adjusted	Multivariable-adjusted^a^
**Antidiabetic drugs, except insulin and GLP-1 receptor agonists**	108 (9.5%)	68 (6.9%)	2.6 (0.3–4.9)	1.40 (1.04_1.90)	1.37 (1.01–1.86)	1.29 (0.91–1.84)
**Insulin or GLP-1 receptor agonist**	30 (2.6%)	14 (1.4%)	1.2 (−0.01–2.5)	1.89 (1.00–3.57)	1.76 (0.93–3.33)	1.02 (0.50–2.06)
**Lipid-lowering drugs**	11 (1.0%	22 (2.2%	−1.3 (−2.5–-0.2)	0.44 (0.21–0.91)	0.41 (0.20–0.86)	0.61 (0.26–1.45)
**H2 blocker or PPI**	11 (1.0%)	15 (1.5%	−0.5 (−1.6–0.4)	0.65 (0.30–1.42)	0.64 (0.29–1.41)	0.51 (0.20–1.25)
**Antinauseants**	11 (1.0%	8 (0.8%	0.2 (−0.7–1.0)	1.22 (0.49–3.02)	1.32 (0.53–3.31)	2.35 (0.79–6.97)
**Antihypertensives**	20 (1.8%)	22 (2.2%	−0.5 (−1.7–0.7)	0.80 (0.44–1.46)	0.79 (0.43–1.45)	0.86 (0.43–1.75)
**Laxatives**	9 (0.8%	15 (1.5%	−0.7 (−1.8–0.2)	0.53 (0.23–1.22)	0.56 (0.25–1.28)	0.45 (0.17–1.15)
**Probiotics**	8 (0.7%)	8 (0.8%)	−0.1 (−1.0–0.7)	0.89 (0.33–2.37)	0.92 (0.34–2.46)	1.03 (0.31–3.42)
**Heart failure drugs**	7 (0.6%)	4 (0.4%)	0.2 (−0.5–0.9)	1.58 (0.46–5.41)	1.64 (0.48–5.60)	1.40 (0.29–6.78)
**Acetaminophen**	6 (0.5%	15 (1.5%	−1.0 (−2.0–-0.1)	0.35 (0.14–0.91)	0.34 (0.13–0.88)	0.28 (0.09–0.85)
**Antihistamines**	5 (0.4%)	10 (1.0%	−0.6 (−1.5–0.2)	0.44 (0.15–1.28)	0.38 (0.13–1.12)	0.43 (0.12–1.57)
**Fluoroquinolones**	5 (0.4%)	5 (0.5%)	−0.1 (−0.8–0.6)	0.88 (0.26–3.05)	0.83 (0.24–2.91)	0.78 (0.19–3.24)

^a^Adjusted for age; male sex; smoking status; use of alcohol, antihypertensives, antidiabetic drugs (except insulin), insulin, lipid-lowering drugs, hypouricemic agents, and steroids (except topical steroids); hypertension; dyslipidemia; myocardial infarction; chronic heart failure; cancer; hyperuricemia; and hospitalization.

H2, histamine 2 receptor; PPI, proton pump inhibitor

## Discussion

In this retrospective cohort study, we evaluated the incidence of treatment-related events among new users of imeglimin and sitagliptin, with particular focus on patients aged ≥65 years. Cox proportional hazards analysis showed no significant difference in hypoglycemia risk between the two groups. Notably, no significant difference in hypoglycemia risk was observed between imeglimin and sitagliptin, despite a higher proportion of insulin use and greater clinical severity in the imeglimin group, which is consistent with the favorable safety profile reported in previous studies. However, imeglimin was associated with a markedly higher risk of gastrointestinal symptoms, including nausea, diarrhea, vomiting, and abdominal discomfort, than sitagliptin. The approximately 1.5-fold higher discontinuation rate observed with imeglimin likely reflects the increased incidence of gastrointestinal events and suboptimal glycemic control. Although some confidence intervals were wide owing to the limited sample size, these findings underscore the need for careful monitoring of gastrointestinal adverse events during the first 4 months of imeglimin therapy, particularly in older patients with type 2 diabetes.

This study is among the first real-world investigations to directly compare hypoglycemia risk between imeglimin and sitagliptin. Previous RCTs [21,23,30–36] evaluating each agent versus placebo reported no significant increase in hypoglycemia risk. The present findings are consistent with these reports, suggesting that imeglimin has a hypoglycemia safety profile comparable to that of sitagliptin.

Importantly, although the imeglimin group included a significantly higher proportion of insulin users (19.5% vs. 5.9%) than the sitagliptin group, hypoglycemia risk remained similar between groups. This observation further supports the favorable safety profile of imeglimin, even among patients with more advanced disease or those requiring complex treatment regimens.

Regarding gastrointestinal tolerability, prior studies [21,30] have reported a 1.3- to 1.5-fold increased risk of gastrointestinal symptoms with imeglimin compared with placebo, whereas meta-analyses [15] indicate that sitagliptin is associated with risks of nausea and diarrhea comparable to placebo. In contrast, the present real-world analysis demonstrated a five-fold higher risk of gastrointestinal symptoms among imeglimin users than among sitagliptin users. This finding was supported by sensitivity analyses restricted to monotherapy users. Although the multivariable-aHR for antinauseant use showed a relatively high point estimate (aHR: 2.35), the association was not significant, and the CI was wide, reflecting limited precision. However, the absolute incidence of antinauseant use was low in both the imeglimin (1.0%) and sitagliptin (0.8%) groups, corresponding to an absolute risk difference of only 0.2%. These findings indicate that while there may be a slight trend toward increased use of symptomatic medications, the absolute number of patients requiring antinauseant therapy was quite small in both groups, suggesting that these gastrointestinal events were generally manageable.

This study has several methodological strengths. Primary data collection by community pharmacists enabled detection of mild events, such as soft stools and abdominal distension, which are often underreported in large claims databases. In addition, the inclusion of detailed lifestyle data (e.g., smoking status, alcohol consumption, and OTC medication use) enhanced internal validity through more comprehensive adjustment. Generalizability is supported by the participation of approximately 10% of pharmacies affiliated with the JPA nationwide [37]. Furthermore, our sensitivity analyses using Firth’s penalized likelihood approach confirmed the robustness of the estimates despite the limited number of events.

This study has several limitations that warrant consideration. First, there are concerns regarding the potential for selection and attrition biases. Because new users were identified by pharmacists according to three predefined eligibility criteria, one of which required at least one follow-up visit, we could not determine the exact number of patients excluded owing to a single pharmacy visit (lost to follow-up). If patients in either group dropped out early owing to intolerable adverse events, this could introduce attrition bias. Additionally, the proportion of patients excluded because they were “switchers” between the study drugs was substantially higher in the imeglimin group (12%) than in the sitagliptin group (0.9%). This suggests that the imeglimin group may have included patients with more severe or refractory diabetes, and residual confounding by indication may have remained despite multivariable adjustments. Second, the non-significant difference in hypoglycemia risk, coupled with the wide CI (0.20–2.22), suggests limited precision, likely owing to the limited number of hypoglycemic events. Third, owing to the nature of our data collection approach, we only obtained information on the concomitant use of non-insulin antidiabetic medications or insulin rather than the specific names of the drugs. A nationwide Japanese study reported that while the use of GLP-1 receptor agonists is extremely low (<1%), biguanides (metformin) are prescribed in approximately 16% of patients [12]. Although our sensitivity analyses restricted to monotherapy users showed consistent results, we cannot completely rule out the possibility of residual confounding by specific concomitant medications in the overall cohort. In particular, since gastrointestinal events represent the most common side effects of metformin [38], the concomitant metformin use may have influenced the overall incidence observed for these gastrointestinal events. Fourth, the 4-month follow-up was shorter than that (9–13 months) typically observed in certain RCTs [31,34], potentially limiting the detection of long-term adverse outcomes. Fifth, the absence of longitudinal laboratory data (e.g., HbA1c) precluded comparative assessment of glycemic efficacy. Sixth, although a higher incidence of elevated blood glucose was observed in the imeglimin group, it is unclear whether this finding reflects confounding by indication, owing to more advanced or refractory disease, or clinical factors related to medication switching or treatment discontinuation. Seventh, incomplete event capture could occur if patients used multiple pharmacies; this risk was mitigated by restricting the analysis to “repeat users” of participating pharmacies. While awareness of imeglimin as a novel agent may have influenced reporting, the new-user design in both cohorts likely minimized such disparities. Finally, while sitagliptin was selected as a representative comparator owing to its highest prescription rate in Japan, it is difficult to attribute these findings to a class effect of DPP-4 inhibitors. Although DPP-4 inhibitors share a common pharmacological mechanism, individual agents may exhibit slightly different safety profiles, and further studies involving other DPP-4 inhibitors are required to confirm class-wide comparative safety against imeglimin.

## Conclusion

This study revealed no significant difference in the risk of hypoglycemia between imeglimin and sitagliptin. However, imeglimin use was associated with a higher incidence of gastrointestinal adverse events, with the absolute risk difference closely approaching our prespecified threshold of 10% for clinical relevance. These findings suggest that patients initiating imeglimin therapy may benefit from careful monitoring for gastrointestinal symptoms, particularly during the first 4 months of therapy. Additional research is warranted to assess whether individuals with a history of gastrointestinal disorders are at an increased risk for these adverse events.

## Supporting information

S1 Table Sensitivity analysis using Firth’s penalized likelihood approach for the incidence of common events among imeglimin and sitagliptin users. ^a^ Adjusted for age; male sex; smoking status; alcohol; use of antihypertensives, antidiabetic drugs (except insulin), insulin, lipid-lowering drugs, hypouricemic agents, and steroids (except topical steroids); hypertension; dyslipidemia; myocardial infarction; chronic heart failure; cancer; hyperuricemia; and hospitalization.
(DOCX)

S2 Table Sensitivity analysis for any add-on drugs using Firth’s penalized Cox regression. ^a^ Adjusted for age; male sex; smoking status; use of alcohol, antihypertensives, antidiabetic drugs (except insulin), insulin, lipid-lowering drugs, hypouricemic agents, and steroids (except topical steroids); hypertension; dyslipidemia; myocardial infarction; chronic heart failure; cancer; hyperuricemia; and hospitalization. H2, histamine 2 receptor; PPI, proton pump inhibitor.
(DOCX)
