# Supplementary material for: Comparative safety of imeglimin versus sitagliptin in Japanese patients with type 2 diabetes: A pharmacy-based retrospective cohort study
Source: PLoS One. 2026 Oct 7;21(10):e0359960. doi: 10.1371/journal.pone.0359960 (PMC13645016; doi:10.1371/journal.pone.0359960)
Supplement: Supplementary file 1 [file pone.0359960.s001.docx]

**Supporting Information**

**S1 Table. Sensitivity analysis using Firth's penalized likelihood approach for the incidence of common events among imeglimin and sitagliptin users.**

| **Common event** | **Imeglimin**  **(n = 1,140)** | **Sitagliptin**  **(n = 990)** | **Absolute risk difference, % (95% confidence interval)** | **Hazard ratio (95% confidence interval)** | | |
| --- | --- | --- | --- | --- | --- | --- |
|  |  |  |  | **Unadjusted** | **Age–sex-adjusted** | **Multivariable-adjusted^a^** |
| **Gastrointestinal symptoms** | 146 (12.8%) | 27 (2.7%) | 9.8 (7.7–12.0) | 4.76 (3.17–7.17) | 4.84 (3.21–7.29) | 5.35 (3.39–8.44) |
| **Nausea** | 41 (3.6%) | 2 (0.2%) | 3.4 (2.3–4.7) | 14.68 (4.03–53.38) | 14.38 (3.94–52.40) | 12.61 (3.32–47.85) |
| **Diarrhea** | 34 (3.0%) | 4 (0.4%) | 2.6 (1.5–3.8) | 6.80 (2.51–18.39) | 6.55 (2.41–17.77) | 7.06 (2.39–20.85) |
| **Constipation** | 14 (1.2%) | 10 (1.0%) | 0.2 (-0.8–1.2) | 1.22 (0.54–2.74) | 1.34 (0.60–3.03) | 1.33 (0.51–3.51) |
| **Abdominal discomfort** | 12 (1.1%) | 3 (0.3%) | 0.8 (0.01–1.6) | 3.18 (0.93–10.80) | 3.39 (0.99–11.63) | 5.36 (1.37–21.05) |
| **Gastrointestinal disorder** | 8 (0.7%) | 3 (0.3%) | 0.4 (-2.8–1.1) | 2.14 (0.58–7.86) | 2.07 (0.56–7.68) | 2.11 (0.47–9.55) |
| **Abdominal distension** | 7 (0.6%) | 2 (0.2%) | 0.4 (-2.1–1.1) | 2.64 (0.59–11.95) | 2.95 (0.64–13.51) | 4.31 (0.75–24.82) |
| **Upper abdominal pain** | 2 (0.2%) | 3 (0.3%) | -0.1 (-0.7–0.4) | 0.63 (0.11–3.70) | 0.61 (0.10–3.63) | 0.82 (0.09–7.41) |
| **Decreased therapeutic response** | 70 (6.1%) | 43 (4.3%) | 1.8 (-0.1–3.7) | 1.43 (0.98–2.10) | 1.31 (0.89–1.92) | 1.14 (0.74–1.76) |
| **Inadequate control of diabetes mellitus** | 23 (2.0%) | 14 (1.4%) | 0.6 (-0.1–1.8) | 1.43 (0.74–2.78) | 1.41 (0.72–2.75) | 1.14 (0.53–2.45) |
| **Increased blood glucose** | 19 (1.7%) | 3 (0.3%) | 1.4 (0.5–2.3) | 4.92 (1.54–15.74) | 4.75 (1.48–15.29) | 4.04 (1.15–14.19) |
| **Increased glycosylated hemoglobin** | 14 (1.2%) | 9 (0.9%) | 0.3 (-0.6–1.3) | 1.36 (0.59–3.13) | 1.26 (0.54–2.92) | 1.30 (0.49–3.49) |
| **Therapy non-responder** | 14 (1.2%) | 3 (0.3%) | 0.09 (0.2–1.8) | 3.65 (1.10–12.12) | 3.30 (0.99–11.03) | 3.43 (0.89–13.23) |
| **Pre-existing condition improved** | 6 (0.5%) | 18 (1.8%) | -1.3 (-2.4–-0.4) | 0.31 (0.13–0.77) | 0.31 (0.12–0.77) | 0.25 (0.09–0.72) |
| **Decreased glycosylated hemoglobin** | 6 (0.5%) | 5 (0.5%) | 0.02 (-0.71–0.70) | 1.05 (0.32–3.45) | 1.16 (0.35–3.83) | 1.06 (0.25–4.55) |
| **Decreased appetite** | 16 (1.4%) | 3 (0.3%) | 1.1 (0.3–2.0) | 4.13 (1.26–13.48) | 4.77 (1.44–15.76) | 2.59 (0.71–9.52) |
| **Increased blood pressure** | 14 (1.2%) | 11 (1.1%) | 0.1 (-0.9–1.1) | 1.11 (0.50–2.44) | 1.18 (0.53–2.61) | 1.28 (0.51–3.26) |
| **Pruritus** | 5 (0.4%) | 10 (1.0%) | -0.6 (-1.5–0.2) | 0.46 (0.16–1.33) | 0.51 (0.18–1.49) | 0.65 (0.18–2.38) |
| **Hypertension** | 4 (0.4%) | 6 (0.6%) | -0.3 (-1.0–0.4) | 0.61 (0.17–2.16) | 0.58 (0.16–2.07) | 0.57 (0.12–2.71) |
| **Dizziness** | 8 (0.7%) | 5 (0.5%) | 0.2 (-0.6–0.9) | 1.36 (0.45–4.13) | 1.59 (0.52–4.86) | 1.47 (0.39–5.58) |
| **Hypoglycemia** | 7 (0.6%) | 7 (0.7%) | -0.1 (-0.9–0.7) | 0.87 (0.31–2.49) | 0.91 (0.32–2.62) | 0.66 (0.19–2.24) |
| **Insomnia** | 5 (0.4%) | 5 (0.5%) | -0.1 (-0.8–0.6) | 0.87 (0.25–3.01) | 0.87 (0.25–3.02) | 0.66 (0.15–2.87) |
| **Increased blood cholesterol** | 4 (0.4%) | 6 (0.6%) | -0.3 (-1.0–0.4) | 0.61 (0.17–2.15) | 0.62 (0.17–2.20) | 0.61 (0.14–2.78) |

^a^ Adjusted for age; male sex; smoking status; alcohol; use of antihypertensives, antidiabetic drugs (except insulin), insulin, lipid-lowering drugs, hypouricemic agents, and steroids (except topical steroids); hypertension; dyslipidemia; myocardial infarction; chronic heart failure; cancer; hyperuricemia; and hospitalization.
