# Supplementary material for: Comparative safety of imeglimin versus sitagliptin in Japanese patients with type 2 diabetes: A pharmacy-based retrospective cohort study
Source: PLoS One. 2026 Oct 7;21(10):e0359960. doi: 10.1371/journal.pone.0359960 (PMC13645016; doi:10.1371/journal.pone.0359960)
Supplement: Supplementary file 2 [file pone.0359960.s002.docx]

**Supporting Information**

**S2 Table. Sensitivity analysis for any add-on drugs using Firth's penalized Cox regression.**

| **Drug** | **Imeglimin (n = 1,140)** | **Sitagliptin (n = 990)** | **Absolute risk difference, % (95% confidence interval)** | **Hazard ratio (95% confidence interval)** | | |
| --- | --- | --- | --- | --- | --- | --- |
|  |  |  |  | **Crude** | **Age–sex-adjusted** | **Multivariable-adjusted^a^** |
| **Antidiabetic drugs, except insulin and GLP-1 receptor agonists** | 108 (9.5％) | 68 (6.9％) | 2.6 (0.3–4.9) | 1.40 (1.03-1.89) | 1.37 (1.01–1.86) | 1.28 (0.90–1.82) |
| **Insulin or GLP-1 receptor agonist** | 30 (2.6％) | 14 (1.4％) | 1.2 (-0.01–2.5) | 1.86 (0.99–3.50) | 1.72 (0.91–3.26) | 0.99 (0.49–2.00) |
| **Lipid-lowering drugs** | 11 (1.0％) | 22 (2.2％) | -1.3 (-2.5–-0.2) | 0.45 (0.22–0.93) | 0.42 (0.20–0.87) | 0.62 (0.26–1.46) |
| **H2 blocker or PPI** | 11 (1.0％) | 15 (1.5％) | -0.5 (-1.6–0.4) | 0.66 (0.30–1.43) | 0.65 (0.30–1.42) | 0.50 (0.20–1.25) |
| **Antinauseants** | 11 (1.0％) | 8 (0.8％) | 0.2 (-0.7–1.0) | 1.20 (0.48–2.97) | 1.32 (0.53–3.31) | 2.30 (0.77–6.88) |
| **Antihypertensives** | 20 (1.8％) | 22 (2.2％) | -0.5 (-1.7–0.7) | 0.80 (0.44–1.47) | 0.79 (0.43–1.46) | 0.85 (0.42–1.73) |
| **Laxatives** | 9 (0.8％) | 15 (1.5％) | -0.7 (-1.8–0.2) | 0.55 (0.24–1.24) | 0.58 (0.26–1.33) | 0.45 (0.17–1.18) |
| **Probiotics** | 8 (0.7％) | 8 (0.8％) | -0.1 (-1.0–0.7) | 0.89 (0.33–2.38) | 0.94 (0.35–2.51) | 1.01 (0.30–3.38) |
| **Heart failure drugs** | 7 (0.6％) | 4 (0.4％) | 0.2 (-0.5–0.9) | 1.51 (0.45–5.11) | 1.62 (0.48–5.53) | 1.41 (0.30–6.65) |
| **Acetaminophen** | 6 (0.5％) | 15 (1.5％) | -1.0 (-2.0–-0.1) | 0.37 (0.15–0.95) | 0.35 (0.14–0.91) | 0.29 (0.10–0.88) |
| **Antihistamines** | 5 (0.4％) | 10 (1.0％) | -0.6 (-1.5–0.2) | 0.46 (0.16–1.28) | 0.40 (0.14–1.16) | 0.45 (0.12–1.65) |
| **Fluoroquinolones** | 5 (0.4％) | 5 (0.5％) | -0.1 (-0.8–0.6) | 0.88 (0.26–3.05) | 0.83 (0.24–2.90) | 0.78 (0.18–3.37) |

^a^ Adjusted for age; male sex; smoking status; use of alcohol, antihypertensives, antidiabetic drugs (except insulin), insulin, lipid-lowering drugs, hypouricemic agents, and steroids (except topical steroids); hypertension; dyslipidemia; myocardial infarction; chronic heart failure; cancer; hyperuricemia; and hospitalization.

H2, histamine 2 receptor; PPI, proton pump inhibitor.
